# Laparoscopic anatomical segment 3 segmentectomy for hepatocellular carcinoma accompanied by hypoplasia of the right hepatic lobe

**DOI:** 10.1093/jscr/rjz213

**Published:** 2019-07-10

**Authors:** Hisoka Yamane, Sachiko Yoshida, Toshihiko Yoshida, Masayasu Nishi, Takashi Yamagishi, Hironobu Goto, Dai Otsubo, Akinobu Furutani, Taku Matsumoto, Yasuhiro Fujino, Masahiro Tominaga

**Affiliations:** Division of Gastroenterological Surgery, Hyogo Cancer Center, Hyogo, Japan

## Abstract

We report a case of laparoscopic anatomical segment 3 segmentectomy for hepatocellular carcinoma (HCC) accompanied by hypoplasia of the right hepatic lobe. An 80-year-old man was admitted with a suspicion of HCC diagnosed by computed tomography during follow-up for thyroid cancer. Dynamic computed tomography showed 40-mm HCC in segment 3 and hypoplasia of the right hepatic lobe with the Chilaiditi sign. We performed laparoscopic anatomical segment 3 segmentectomy. There were no postoperative complications, and the patient was discharged 6 days postoperatively. This procedure can be performed safely and is technically feasible, but special attention should be paid to anatomical alterations to avoid fatal surgical complications.

## INTRODUCTION

Anatomical segmentectomy has an oncological benefit compared with nonanatomical liver resection in the surgical treatment of hepatocellular carcinoma (HCC) [1], because this procedure systematically removes potentially tumor-bearing portal and venous tributaries around malignant lesions [2]. However, even in open liver surgery, anatomical segmentectomy is often more complicated and difficult than major hepatectomy [3].

Over the last decade, laparoscopic hepatectomy has been increasingly performed throughout the world [4]. Although laparoscopic left lateral sectionectomy has been recognized as the standard approach for tumors in segments 2 and/or 3 [5], there have been few reports about laparoscopic segmentectomy for patients with HCC in segment 2 or 3 [6]. Herein, we present a case report of laparoscopic anatomical segment 3 (S3) segmentectomy for HCC accompanied by hypoplasia of the right hepatic lobe.

## CASE REPORT

**Figure 1:**
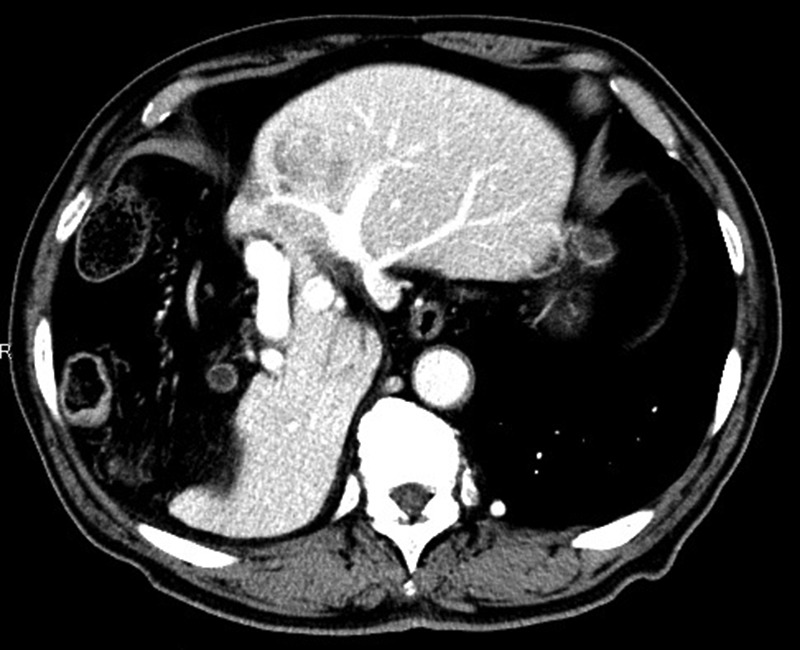
Dynamic contrast-enhanced CT showing a 40-mm nodule in S3 and hypoplasia of the right hepatic lobe with the Chilaiditi sign.

An 80-year-old man was undergoing follow-up because he had undergone surgery for thyroid cancer 2 years previously in the Department of Head and Neck Surgery at our hospital. Dynamic contrast-enhanced computed tomography (CT) during follow-up for thyroid cancer revealed a mass measuring 40 mm in diameter with high attenuation, early-phase enhancement, washout during the late-phase in S3 and hypoplasia of the right hepatic lobe with the Chilaiditi sign, which is a segmental interposition of the colon between the liver and the diaphragm (Fig. 1). Our preoperative diagnosis was HCC, and the clinical stage was T1bN0M0, or Stage IB, in terms of the Union for International Cancer Control classification (eighth edition).

Tests for hepatitis B virus surface antigen and antibodies against hepatitis C virus were negative. Liver function tests were graded as Child-Pugh class A, but the 15-min retention rate for indocyanine green (ICG15) was 19.0%. His serum proteins induced by the absence of vitamin K or antagonist-II measured 670 mAU/mL, and serum alpha-fetoprotein levels was within the normal range. The three-dimensional volume analyzer Synapse Vincent^TM^ (FUJI-FILM Co., Japan) revealed that the left lateral section exhibited compensatory hypertrophy with a volume of 520 ml volume, and 45.2% total liver volume.

**Figure 2:**
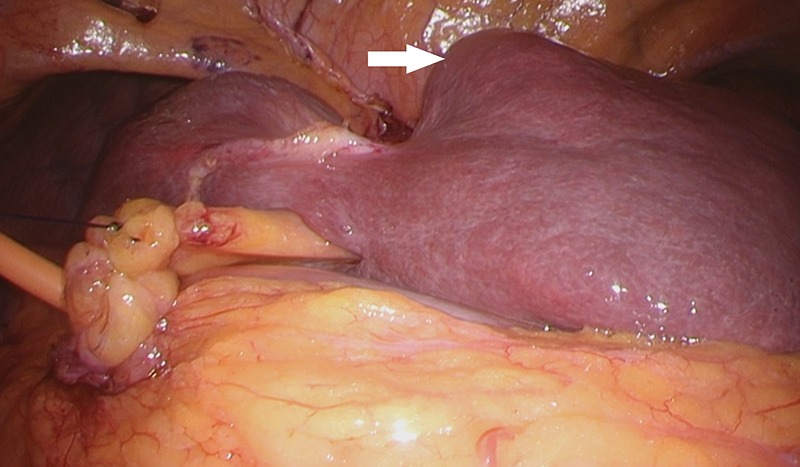
The small right hepatic lobe is not fixed to the diaphragm. The arrow (white) shows the lesion in S3.

**Figure 3:**
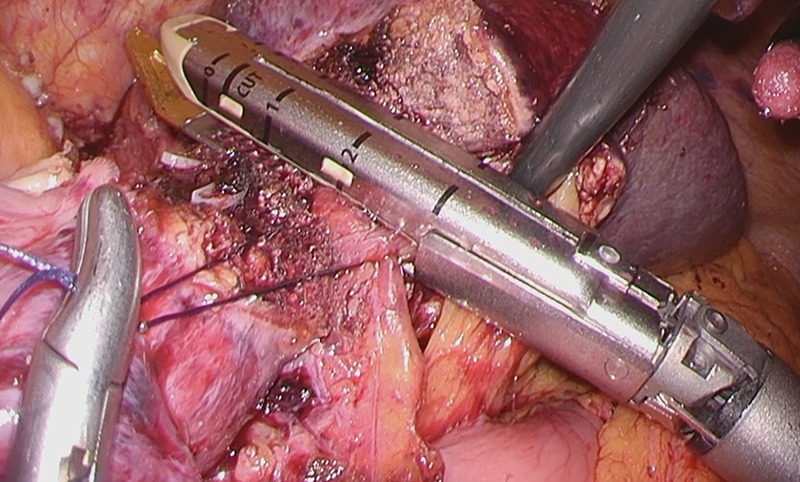
The Glissonian pedicle to S3 was transected using the Signia^TM^ Stapling System.

**Figure 4:**
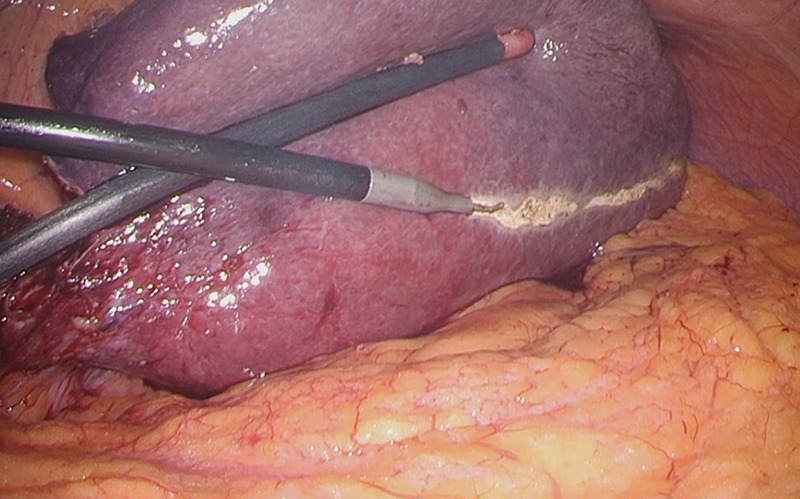
The ischemic margin of S3 was marked using electrocautery.

**Figure 5:**
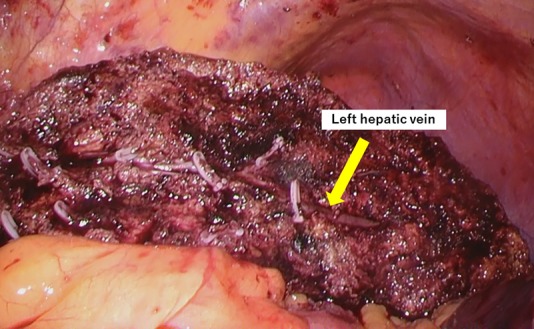
The liver parenchyma was transected along the left hepatic vein.

**Figure 6:**
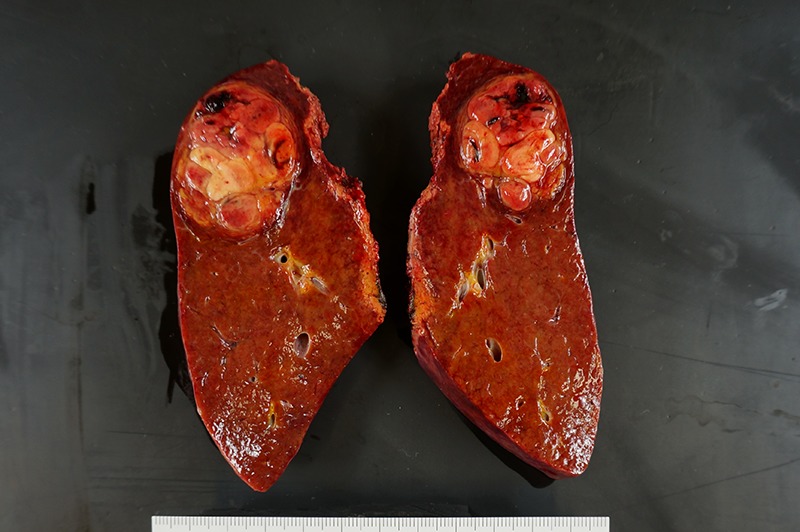
The resected specimen shows a 41-mm simple nodular type of HCC and a 5-mm tumor-free resection margin.

**Figure 7:**
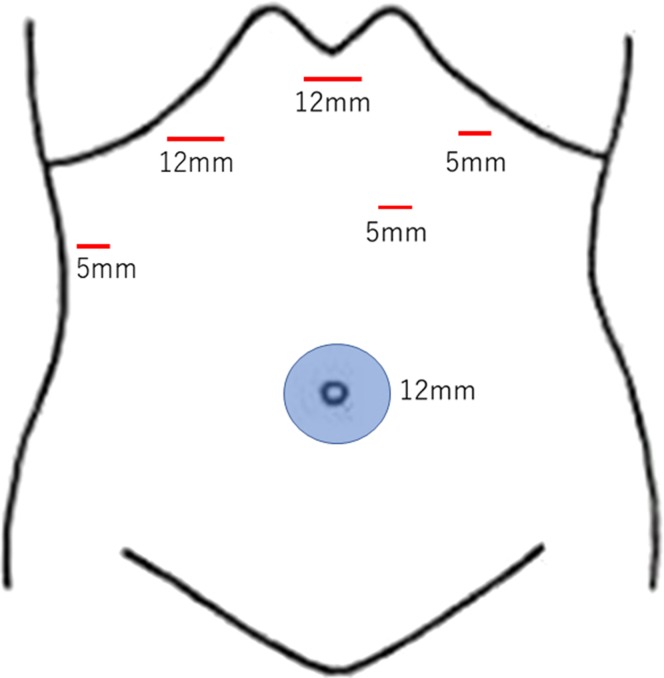
The schema demonstrates the placement of each port.

Considering the small remnant right lobe and abnormal ICG15, we planned to perform laparoscopic anatomical S3 segmentectomy. The procedures were performed using a pressure-controlled carbon dioxide pneumoperitoneum, which was maintained below 12 mmHg. Intraoperative findings showed hypoplasia of the right hepatic lobe and hypertrophy of the left hepatic lobe (Fig. 2). Intraoperative ultrasound (IOUS) was performed to confirm the location of the tumor and its relationship to the adjacent structures. The Glissonian pedicle to S3 was isolated with meticulous dissection and was then transected using the Signia^TM^ Stapling System and Endo GIA^TM^ Curved Tip Reload with Tri-Staple^TM^ Technology 30 mm, Vascular Medium (Covidien, USA) (Fig. 3). The ischemic margin of S3 was marked using electrocautery (Fig. 4). Using the crush-clamp method with a harmonic scalpel (Ethicon, USA), the liver parenchyma was transected along the left hepatic vein (Fig. 5). Pringle’s maneuver was performed by clamping the hepatoduodenal ligament using the tourniquet method for 15 min with following a 5-min release period, and a total of fourteen temporary clamps were performed during parenchymal resection. The resected specimens were removed in a retrieval bag through an umbilical port site. The resected specimen showed a 41-mm simple nodular type of HCC and a 5-mm tumor-free resection margin (Fig. 6). Each port was placed as shown in Fig. 7.

The operative time was 549 min. The estimated intraoperative blood loss was 1000 ml, and a blood transfusion was not required. Postoperative pathological examination showed moderately differentiated HCC measuring 4.1 cm in diameter with a 5-mm tumor-free resection margin. The noncancerous portion of the resected liver confirmed a diagnosis of chronic hepatitis. The patient had a favorable clinical course without any complications, and he was discharged on postoperative day 6. There was no sign of recurrence 6 months after surgery.

## DISCUSSION

Many studies have reported laparoscopic hepatectomy has the advantages of a fast postoperative recovery and decreased wound complications, as well as a technical feasibility and oncological safety comparable to those of open procedure [7]. Moreover, anatomical liver resection has an oncological benefit compared with nonanatomical liver resection in the surgical treatment of HCC [1], because this procedure systematically removes potentially tumor-bearing portal and venous tributaries around malignant lesions [2]. Hence, laparoscopic anatomical monosegmentectomy can be an ideal treatment option for HCC confined to one anatomical segment. However, even in open liver surgery, anatomical segmentectomy is often more complicated and difficult than major hepatectomy, and laparoscopic segmentectomy requires high technical skills and is rarely reported [3]. Although laparoscopic left lateral sectionectomy has already been recognized as the standard approach [5], there have been few reports about laparoscopic segmentectomy for patients with HCC in segment 2 or 3 [6]. We experienced a rare case of laparoscopic anatomical S3 segmentectomy for HCC with hypoplasia of the right hepatic lobe.

According to the studies of Pages *et al.*, morphologic anomalies related to developmental defects can be categorized as follows: agenesis (absence of a lobe that is replaced by fibrous tissue); aplasia (one of the lobes is small and its structure is abnormal, with few hepatic trabeculae, numerous bile ducts, and abnormal blood vessels); or hypoplasia (one of the lobes is small but is normal in structure) [8]. According to this classification, our case is considered hypoplasia. Hypoplasia of the right liver lobe is an extremely rare congenital anomaly and a rare cause of the Chilaiditi sign.

We think that laparoscopic anatomical S3 segmentectomy is the best treatment option to preserve the nontumor-bearing liver parenchyma with a decreased risk of posthepatectomy liver insufficiency in our patient with a small remnant right lobe. Although intraoperative blood loss amount was large, this is because the left lateral section exhibited compensatory hypertrophy and an area of the liver cut surface was big. Laparoscopic anatomical segmentectomy is complicated and technically demanding and should be performed by surgeons with advanced experience both in open and laparoscopic surgery. This procedure can be performed safely and is technically feasible with appropriate use of the Glissonian approach [9] and special attention should be paid to anatomical alterations to avoid fatal surgical complications.

## CONCLUSION

In summary, we reported laparoscopic anatomical S3 segmentectomy for HCC accompanied by hypoplasia of the right hepatic lobe.
